# Hot Gas‐Blowing Assisted Crystallinity Management of Bar‐Coated Perovskite Solar Cells and Modules

**DOI:** 10.1002/smsc.202300069

**Published:** 2023-07-18

**Authors:** Minsung Han, Junseop Byeon, Jihun Jang, Changnyeong Hur, Gabseok Seo, Mansoo Choi

**Affiliations:** ^1^ Department of Mechanical Engineering Seoul National University Seoul 08826 Republic of Korea; ^2^ Global Frontier Center for Multiscale Energy Systems Seoul National University Seoul 08826 Republic of Korea; ^3^ Frontier Energy Solution Corporation Seoul National University Seoul 08826 Republic of Korea

**Keywords:** bar coating, hot gas blowing, large area, perovskite

## Abstract

The bar‐coating technique for perovskite solar cells has been studied as a scalable process in relation to solar cell commercialization. In large‐area bar coating, solvents with the high boiling points like dimethylformamide or *γ*‐butyrolactone have difficulty in obtaining uniform and planar film at room temperature as they have slow evaporation rate. As an alternative, 2‐methoxyethanol is a volatile and polar solvent, which is useful on a bar coating if applied using an air‐blowing method with an air knife. Herein, a hot gas‐blowing method for the fabrication of a perovskite layer to achieve both proper solvent evaporation and high crystallinity is developed. With 75 °C of N_2_ blowing on the bar‐coated perovskite solution, highly crystalline perovskite films with large grains without voids are fabricated, showing excellent optical and electrical characteristics, such as long carrier lifetime, few carrier recombinations, and low trap density. Both small‐area solar cells and large‐area modules show good performance, 20.85% for the solar cell and 15.4% for the solar module. The results indicate that the newly proposed method can equally be applied to the fabrication of large‐area solar cells toward commercialization.

## Graphical abstract

A high‐quality 2‐methoxyethanol‐based perovskite layer of large‐area bar coating is manufactured by applying the hot gas‐blowing method, which contributes to solvent evaporation of the perovskite precursor solution and induces effective crystallization, resulting in high‐performance perovskite solar cells and solar modules showing excellent optical and electrical properties of the light‐absorbing layer.

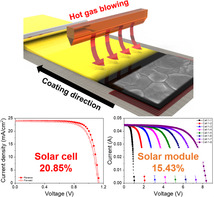

## 1 Introduction

Lead halide perovskite materials are widely used in a variety of devices such as detectors, LED, and flexible electronics, owing to their outstanding optoelectronic properties.^[^ ^1^, ^2^, ^3^, ^4^ ^]^ Especially, in the field of photovoltaics, expectations that perovskite solar cells (PSCs) will serve as an alternative for conventional thin film photovoltaics have rapidly soared, given their certified power conversion efficiency (PCE) exceeding over 25% in less than two decades.^[^ ^5^, ^6^ ^]^ These outcomes are, however, unfortunately, still far from the solar market's required performances of the perovskite solar modules to be commercialization, especially given a large PCE gap compared to those of state‐of‐the‐art devices on the lab scale.^[^ ^7^ ^]^ Scalable‐coating techniques to ensure uniform coverage, reproducibility, and high crystallinity of perovskite films are required to commodify PSCs in the real market. In an effort to fabricate high‐quality large‐area films, many techniques for scalable coatings have been investigated such as bar/blade, slot‐die, and vapor phase deposition methods since the conventional spin‐coating method is limited to fabricating films over a large area.^[^ ^8^, ^9^, ^10^, ^11^, ^12^, ^13^, ^14^ ^]^ Among various scalable coating techniques, the bar‐coating method that uses an air knife is considered as a cost‐effective and facile technique owing to the characteristics of ambient‐air processable printing and a low loss of the precursor solution.^[^ ^15^ ^]^ Large‐area perovskite solar modules fabricated by bar coating have achieved remarkable results, demonstrating the feasibility of mass production.^[^ ^16^, ^17^ ^]^

With regard to large‐area coatings of perovskite films with a precursor solution, the selection of the solvent is crucial to ensure uniform coverage of perovskite film, as the drying kinetics of the solvent will significantly affect the nucleation and crystal growth mechanism.^[^ ^18^, ^19^, ^20^ ^]^ Unlike the spin‐coating technique, which enables rapid solvent extraction by an antisolvent method, scalable meniscus coatings with blade and bar coating techniques do not use the anti‐solvent method, making rapid extraction of the solvent challenging and complicating the selective evaporation of the solvent.^[^ ^20^, ^21^ ^]^ Although blowing air and heating a substrate can be utilized to support evaporation of the solvent, commonly used polar solvents such as *N*,*N*‐dimethylformamide (DMF), *γ*‐butyrolactone (GBL), and dimethyl sulfoxide (DMSO) have a high boiling point and low volatility at room temperature, hindering the evaporation of the solvents rapidly and selectively.^[^ ^17^, ^22^ ^]^ Because delayed solvent evaporation can lead to the creation of very large islands in, for instance, a crystallite form but with poor surface coverage, it is necessary to use a solvent with high volatility.^[^ ^17^, ^18^, ^22^ ^]^ Among the various feasible solvents, 2‐methoxyethanol (2ME) has emerged as a substitute to overcome the late evaporation in meniscus coatings. Compared to polar solvents conventionally used thus far, 2ME has a low boiling point, high volatility, a low donor level, and high wettability with substrates.^[^ ^22^, ^23^ ^]^ For these reasons, 2ME is regarded as a suitable solvent for a scalable meniscus coating capable of playing a major role in bringing the commercial realization of PSCs closer. Several studies have demonstrated perovskite solar modules via a scalable bar‐coating method using 2ME as a solvent. Huang et al. reached PCE of 15.86% in a MAPbI_3_‐based flexible perovskite solar mini‐module with an active area of 42.9 cm^2^.^[^ ^24^ ^]^ Seok et al. also demonstrate an efficient FAPbI_3_‐based perovskite solar module with certified PCE of 17.53% in an aperture area of 31 cm^2^.^[^ ^17^ ^]^

Along with the appropriate solvent for a scalable coating, it is considered that perovskite crystallinity is important to fabricate efficient perovskite solar devices. Except for methods related to changing materials, such as replacing cations/anions and/or adding additives, many researchers have attempted to devise technical methods that improve the perovskite crystallinity by changing a certain process, such as by‐high temperature annealing for a short time, solvent annealing, and the hot‐casting method.^[^ ^25^, ^26^, ^27^ ^]^ Among them, the hot‐casting method has been widely explored in attempts to realize the fabrication of perovskite film with large grain and high crystallinity.^[^ ^27^, ^28^, ^29^ ^]^ The hot‐casting method takes advantage of the relatively rapid evaporation of the solvent by preheating the precursor solution or the substrate at a certain high temperature, leading to the formation of a film with a low defect density.^[^ ^27^, ^29^ ^]^ The method can also be applied to fabricate a two‐dimensional (2D) perovskite layer because it helps a 2D crystal plane to have a preferred orientation.^[^ ^30^, ^31^ ^]^ However, in scalable coating methods, the hot‐casting method generally has been performed when the perovskite film was fabricated using a precursor solution based on a solvent with a high boiling point.^[^ ^13^, ^27^, ^29^, ^32^ ^]^ This implies that the hot‐casting method is rarely applied to fabricate perovskite films based on a volatile solvent via a scalable‐coating method because the method makes it difficult to control the evaporation rates of the solvent, leading to incomplete formation of the perovskite film. It would be challenging to set the optimum temperature to obtain a hot‐casting effect because volatile solvents such as 2ME and acetonitrile (ACN) have a low boiling point and high vapor pressure at room temperature compared to a commonly used solvent such as DMF and GBL.^[^ ^22^, ^33^, ^34^, ^35^ ^]^ Thus, it is necessary to develop a technology that can achieve a similar effect to that of hot‐casting method by using a solvent with a low boiling point amenable to scalable coating processes.

There are previous studies that used a hot air for fabrication of the PSCs.^[^ ^36^, ^37^, ^38^ ^]^ Su et al. applied a hot air blowing (HAB) method for fabrication of PSCs via ultrasonic spraying process. After perovskite precursor solution was coated on the substrate, they used hot air to evoke solvent evaporation and nucleation with comparison of a heating the substrate method. As a results, high‐quality perovskite films were obtained by using the HAB method and with a MAPbI_3‐x_Cl_x_ composition, 13.5% (0.13 cm^2^) and 9.8% (1 cm^2^) PCEs could be achieved as the champion values.^[^ ^36^ ^]^ Another work, from Mali et al., also showed a dynamic hot‐air‐assisted (DHA) system for fabrication of inorganic PSCs; BaI_2_:CsPbI_2_Br. Compared to the case without a hot air, the perovskite layer with the hot air showed higher power conversion efficiencies in small‐area and large‐area PSCs; 14.85% in 0.09 cm^2^ and large area 13.78% in 1 cm^2^.^[^ ^37^ ^]^ These works showed excellent results that the hot air was contributed to produce high‐quality perovskite films. However, those works were limited only to small‐area fabrication up to 1 cm^2^ because the air gun or nonscalable‐coating methods (i.e., spin coating) used in the papers are not suitable for large‐area processes. Recently, Dai et al. applied the hot gas in the blade coating method to fabricate perovskite tandem modules, but the maximum area of devices is 14.3 cm^2^, still showing the result of small area fabrication.^[^ ^38^ ^]^ For the commercialization of PSCs, it is necessary to develop a scale‐up technology to manufacture high‐performance PSCs in a large area.

Herein, we developed a practical technique that can increase the crystallinity of perovskite, offer uniform coverage, and enhance the performance of PSCs by introducing a hot gas blowing (HGB) method during the bar coating procedure. The blowing gas temperature plays an important role in controlling the drying kinetics of the solvent and the grain growth mechanism based on the volatile solvent 2ME. In this case, blowing N_2_ gas at a certain temperature is applied to regulate the evaporation rate of 2ME after blade coating is carried out, leading to a morphological change in the perovskite film. We found that the temperature of the blowing gas significantly influences the lifetime of photo‐generated carriers, proved by spatial photoluminescence (PL) mapping. Electronic band alignment and the Pb/I ratio were investigated to more closely elucidate the change of the work function (WF) in perovskite films originating from defect formation arising during the coating method assisted by HGB. We demonstrated 2ME‐based PSCs that achieve a PCE of 20.85% under 75 °C hot‐gas‐blowing conditions (denoted as HGB75). To validate the possibility of realizing the perovskite photovoltaics suitable for commercialization, we fabricated a perovskite solar module 5 × 5 cm^2^ in size that showed a PCE of 15.4%. Our technique is the first method to use the HGB for large‐area fabrication without the preheating of a substrate while effectively enhancing the quality of a perovskite solar module in a scalable coating.

## 2 Results and Discussion

**Figure 1:**
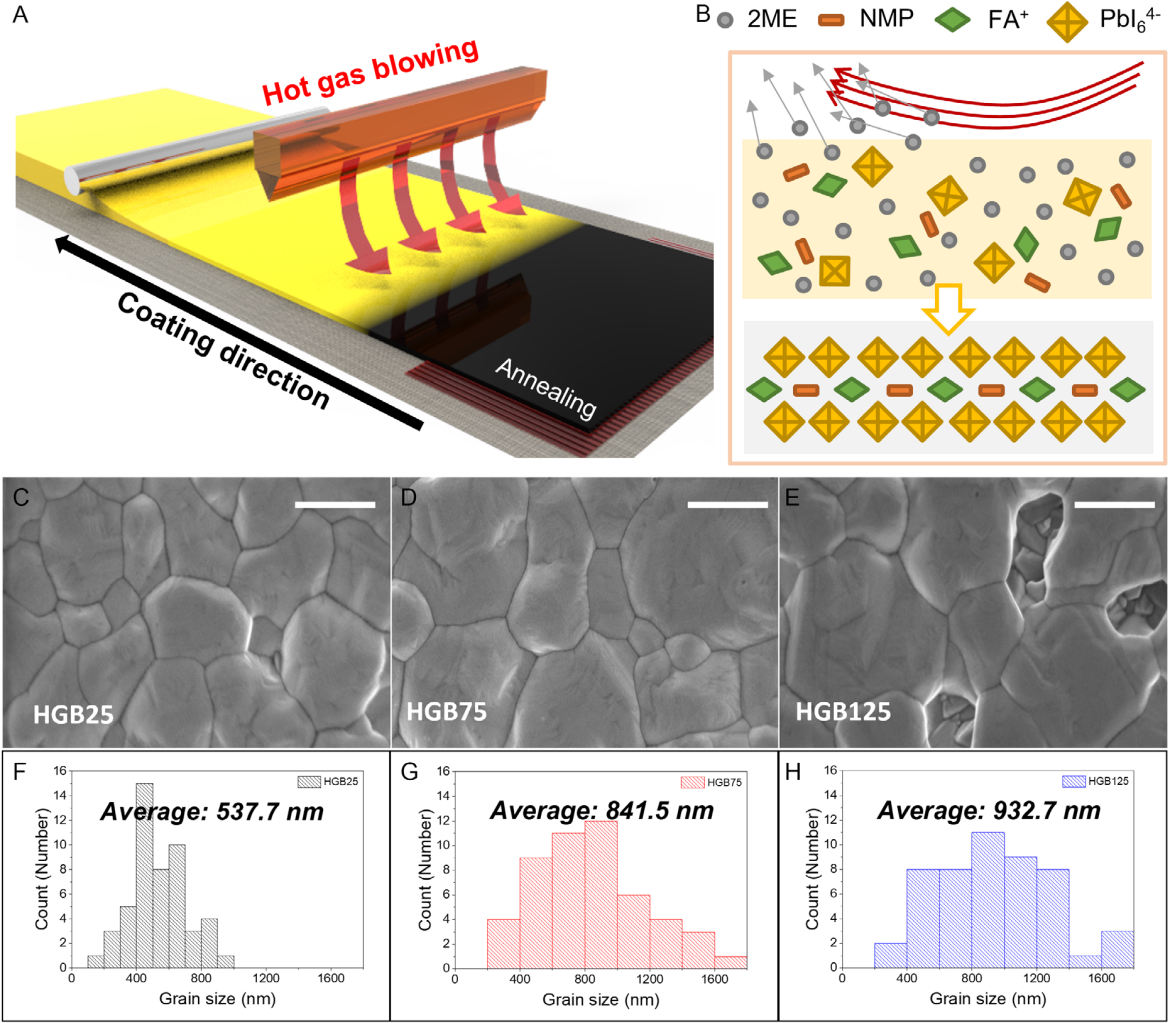
Hot gas‐blowing‐assisted bar‐coated perovskite films. A) Schematic images of hot gas blowing for bar‐coated large area perovskite films: first, thinning the thickness of the solution layer as the bar passes. Second, an intermediate phase is formed through gas blowing. Last, a perovskite layer is formed through annealing treatment. B) Mechanism of solvent evaporation (2‐methoxyethanol) to form an intermediate layer via hot gas blowing. C–E) SEM images of hot gas‐blowing temperature‐dependent perovskite film surface morphologies. Scale bars: 500 nm. F–H). Histogram of perovskite grain size and their mean values of different gas‐blowing temperatures 25, 75, and 125 ºC (HGB25, HGB75, and HGB125), respectively.

We demonstrate that bar coating with the HGB system can enhance the perovskite crystallinity and facilitate the fabrication of a conformal thin film, which is practically applicable to a scalable coating technique. The main idea of this system is to employ an air knife with N_2_ gas at a specific temperature. The system not only improves crystallinity originating from the effect of hot casting but also prevents side effects that may arise when using the highly volatile 2ME solvent and applying the hot casting method at the same time. **Figure** **1**A illustrates the process of bar coating assisted by blowing hot gas. In the system, the 2ME‐based perovskite precursor diffused while the bar moved on the substrate and maintained a constant spatial spacing with the substrate. N_2_ gas at a certain temperature flows over bar‐coated 2ME‐based precursor on the substrate within a short time, evaporating some amount of the solvent and leaving the perovskite intermediate phase, as shown in Figure 1B. (In the system, the 2ME‐based precursor diffused while the rod moved and maintained a constant spatial spacing with the substrate.)

Then, the substrate is annealed to completely evaporate the remained solvent and induce grain growth. The quality of the fabricated perovskite film and full coverage of the film without pinholes is imperative to ensure the high performance of PSCs. To confirm the morphology of the perovskite film through the system devised here, scanning electron microscope (SEM) measurements were carried out. Figure 1C–E shows SEM images of perovskite films fabricated by employing the HGB system depending at the blowing gas temperature of 25, 75, and 125 °C (HGB25, HGB75, and HGB125), respectively. Note that the hot blowing gas at 25 °C is similar to conventional bar coating with an air knife using ambient air. While formamidinium (FA)‐based perovskite crystal fabricated via HGB25 and HGB75 fully covers the glass substrate with a good morphology, perovskite film fabricated under the HGB125 condition shows nonuniform coverage with voids and pinholes. Since the boiling point of 2ME is around 125 °C and given that 2ME easily evaporates under the 125 °C process condition, the poor coverage may be due to the excessively high evaporation rate of the solvent compared to the rate of mass transfer for grain growth.^[^ ^23^, ^39^ ^]^ To clarify the effect of the blowing gas temperature on the grain size of the perovskite, we calculated the grain size from SEM images using ImageJ software and acquired a statistical histogram with respect to the blowing gas temperature. As shown in Figure 1F, the average grain size of perovskite film in the HGB25 case is 537.7 nm. However, the grain size of perovskite film fabricated by the HGB technique increased significantly to 841.5 and 932.7 nm via HGB75 and HGB125 condition, respectively (see Figure 1G,H). These results indicate that heat transfer from the HGB served not only to evaporate solvent but also to facilitate grain growth. The effect of HGB was also observed in a perovskite film fabricated by hot casting method.^[^ ^27^, ^29^ ^]^ The system developed here can realize advantages similar to those of hot casting, preventing a nonuniform morphology induced by the fast evaporation of the solvent, as shown in Figure S1, Supporting Information.

To understand the effect of the blowing gas temperature on the grain size, we utilized classical nucleation theory, as indicated by Equation ((1), (2))–(3).^[^ ^40^, ^41^ ^]^ The nuclei, which have enough energy to be stabilized in solution, can selectively continue to grow according to a thermodynamic model.

(1) $$\Delta G_{T}\;=\;\Delta G_{V}\;+\;\Delta G_{S}$$

(2) $$\Delta G_{V}\;=\;\frac{4}{3}\pi r^{3}\Delta g_{V}$$

(3) $$\Delta G_{S}\;=4\pi r^{2}\gamma$$

where Δ*G*_V_ is the change of the volume energy and Δ*G*_S_ is a change of the surface energy. Δ*g*_V_, *γ*, and r indicate the change of the bulk‐Gibbs energy per volume, the surface tension per unit area, and the particle radius in the solution, respectively. Thus, the change of the total Gibbs energy must have a critical point for stabilization, and the radius of the nuclei corresponding to the critical point of the total Gibbs energy can represent a critical radius at which nuclei do not dissolve in the solution, as shown in Figure S2a, Supporting Information. Hence, nuclei with a radius greater than the critical radius (*r*_c_) can be stable in the solution while nuclei with a radius smaller than the critical radius are likely to redissolve into the solution spontaneously. Thus, *r*_c_ can be considered as the criterion denoting the minimum radius at which the phase can be maintained and growth can continue. Because the energy distributions of the nucleus can be expressed as a Boltzmann distribution based on statistical thermodynamics, the probability density of nuclei with energy exceeding the critical Gibbs energy is increased as the temperature of the system increases.^[^ ^41^, ^42^ ^]^

Blowing gas at a temperature of 25 °C could not transport enough energy to overcome the critical Gibbs free energy in the wet film during the bar coating procedure. Thus, nucleation of the perovskites rarely occurs because many embryo does not have enough energy and disintegrated into the solution under the natural blowing conditions, as shown in Figure S2b, Supporting Information. On the other hand, in the 75 °C blowing condition, perovskite nuclei which exceed the critical radius undergo growth immediately from the perovskite seeds since enough energy is supplied during the annealing process. In Figure S2c, Supporting Information, the formation of seeds in the wet film facilitates the diffusion of the solute and the grain growth process. Similar effects were observed in previous studies of a self‐seeding growth method.^[^ ^43^, ^44^ ^]^ In the 125 °C condition, excess energy of the blowing gas at a high temperature causes extremely fast evaporation of the solvent, impeding the mass transport of the solute in the wet film. This leads to the formation of pinholes and voids as previously discussed and as shown in Figure 1E.

To observe the effect of the HGB on the crystallization of perovskite films, we investigated the characteristics of perovskite films before crystallization was fully completed. Instead of annealing the perovskite film for a total of 20 min as described in Experimental section, perovskite films after gas blowing with different temperatures were annealed for 7 s to confirm a more temporary change. Several measurements were conducted and summarized in Figure S3 and S4, Supporting Information. In Figure S3a, Supporting Information, in SEM measurements, when sufficient annealing is not experienced, an incomplete perovskite film layer is formed because the crystallization process is not completed.^[^ ^45^ ^]^ Many pinholes seen in the perovskite films also appear to be caused by uncontrolled late solvent evaporation. However, it can be clearly seen that as the temperature of the blowing gas increases, the non‐alpha phase state (delta phase or adduct‐related complex) decreased and perovskite grains are formed. For a more accurate comparison, A SEM image was obtained for the HGB25 sample after annealing for 3 s, which is shorter than 7 s, and the proportion of the non‐alpha phase state was much higher. In HGB75 and HGB125 samples, the proportion of the non‐alpha phase states decreased and the crystalline state gradually increased. However, in HGB125, it was confirmed that many voids were already generated, which may be due to too fast solvent evaporation by excessive heat. This change was once again confirmed crystallographically through X‐ray diffractometer (XRD) measurements in Figure S3b, Supporting Information. The more the alpha phase of the perovskite was found in the SEM image, the stronger the peak of the alpha phase (at 13.9º) was found in the XRD measurement. Consequently, the HGB has a significant effect on the beginning step of crystallization of perovskite films.

To further analyze the changes in the film, UV–vis absorbance and PL were measured, as shown in Figure S4, Supporting Information. Especially in the absorbance measurement, we conducted an additional experiment to check the effect of HGB without any post‐annealing. Absorbance was measured after gas blowing on perovskite precursor solution coated on the substrate at each temperature for 1 min without post‐annealing on a hot plate and expressed in Figure S4a, Supporting Information, as open symbols. Even after gas blowing on the film for a very long time, no absorption in the wavelength range near 800 nm was found, which is related to the alpha phase of the perovskite. This may be mainly due to the material properties of FA‐based perovskite changing to alpha phase at 150 °C. Rather than, we decided that it would be meaningful to investigate the initial crystallization process from an in situ perspective and compare the differences with the post‐annealing even for a very short time, and the absorbance was expressed in Figure S4a, Supporting Information, as filled symbols. Since the complete annealing process was not performed, the absorbance by alpha phase of the perovskite layer was much lower than the normal perovskite layer (Figure 3A). However, as can be seen in the inset image in Figure S4a, Supporting Information, the absorbance near 800 nm was improved as the gas‐blowing temperature increased, which indicates that the proportion of alpha phase of perovskite increased. In Figure S4b, Supporting Information, steady‐state PL of all samples showed blueshifted PL peaks under 800 nm compared to fully annealed samples (Figure 3B), which is due to the small grain size of perovskite by incomplete annealing period.^[^ ^46^ ^]^ For the HGB25 sample, there is two main peaks; an perovskite peak near 800 nm with weak intensity and a non‐perovskite peak near 650 nm, which is showed in the SEM image (Figure S3a, Supporting Information). As the blowing temperature increases (HGB75 and HGB125), the PL intensity of the non‐perovskite side decreases and the intensity of alpha phase of perovskites becomes stronger. Eventually, we confirmed the effect of the gas‐blowing temperature on perovskite crystallization by observation of the transient change of perovskite films via various measurements.

**Figure 2:**
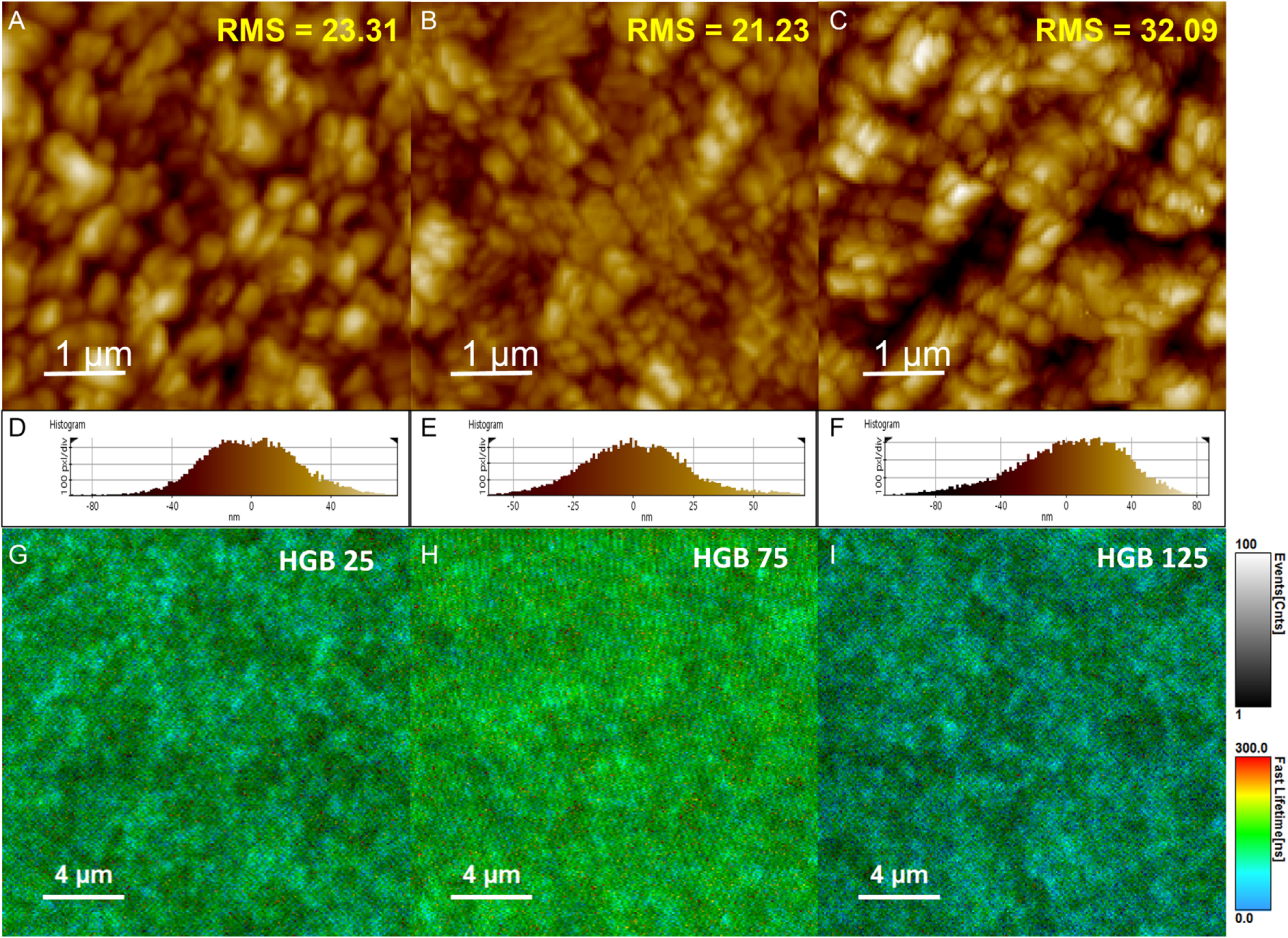
Characteristics of perovskite films. A–C) Topography images of perovskite surfaces of HGB25, HGB75, and HGB125. Root mean square (RMS) roughness values are indicated. Scale bar: 1 μm. D–F) Roughness graphs of different gas‐blowing temperatures. G–I) Photoluminescence mapping on perovskite surfaces of different gas‐blowing temperatures (HGB25, HGB75, and HGB125, respectively). Scale bar: 4 μm.

To gain further insight into the morphological changes of the perovskite film in relation to the blowing gas temperature, a topography image was obtained using atomic force microscopy (AFM). **Figure** **2**A–C shows typical AFM images of the perovskite film fabricated with the HGB25, HGB75, and HGB125 conditions. The grain size of the perovskite film in each condition is fairly consistent with the results obtained from the SEM images. Furthermore, we confirm the surface roughness of each perovskite film shown in Figure 2D–F. The films fabricated by the HGB25 and HGB75 conditions, which have uniform coverage, show similar values of the surface roughness of 23.31 and 21.23 nm, respectively. In contrast, the surface roughness of the perovskite film in the HGB125 condition (32.09 nm) was greater, possibly due to formation of voids and pinholes, in accordance with results in Figure 1E. Detailed film roughness data are described in Table S1, Supporting Information. We also investigated the electronic transport properties of the surface depending on the blowing gas temperature by conductive AFM (c‐AFM), as shown in Figure S5, Supporting Information. Morphology mapping and current spectra of controlled perovskite films are shown in Figure 2A. The c‐AFM image of HGB25 and HGB75 film displayed homogeneous current intensity distributions but showed existence of low current intensities depending on the morphological roughness in some areas. In contrast, in HGB125, all the monitored area was inhomogeneous due to the large roughness of the perovskite crystalline, bottom photocurrent spectra indicated flat and prominent spectra. We found spatial morphology plays an important role in electrical conductivity of perovskite film. Surface roughness accompanied by voids can hinder charge transfer to the charge transporting layer, resulting in high current density–voltage (*J–V*) hysteresis and poor performance.^[^ ^47^, ^48^, ^49^ ^]^ From this result, we can estimate perovskite solar cell using the film by different HGB condition would show different *J–V* characteristics for each device (we will discuss later).

To dig into the microscopic carrier dynamics, we used fluorescence lifetime imaging microscopy. Fluorescence lifetime imaging microscopy (FLIM) is a specialized technique for visualizing the lifetime of photogenerated charge carriers across the film.^[^ ^50^ ^]^ Through the measurement, the lifetime corresponding to the perovskite location and the uniformity of the carrier lifetime across the entire film can be obtained. The changes in the lifetimes of photogenerated charge carriers with different gas temperatures are presented in Figure 2G–I. Blue and dark green indicate regions with short lifetimes while light green and red represent locations with long lifetimes of the charge carriers. The FLIM results reveal that perovskite film in the HGB75 condition exhibited uniform and long PL lifetimes, showing a light green color in Figure 2H. On the other hand, in the HGB25 and HGB125 cases, dark green regions indicate a shorter lifetime, occupying most of the area in the image. Specifically, for the HGB125 condition, dark regions in some parts of the image can be observed, indicating the formation of voids and pinholes, as shown in Figure 2I. This result is consistent with the SEM and AFM images in Figure 1C–E and 2A–C, respectively. Longer carrier lifetimes can be obtained when radiative recombination is a dominant process with low defect density in the film, leading to an enhancement of *V*_OC_ in the device.^[^ ^51^, ^52^ ^]^ From the results shown in Figure 2, we found that hot‐blowing gas can not only enlarge the grain size with high crystallinity but also improve the carrier lifetime.

**Figure 3:**
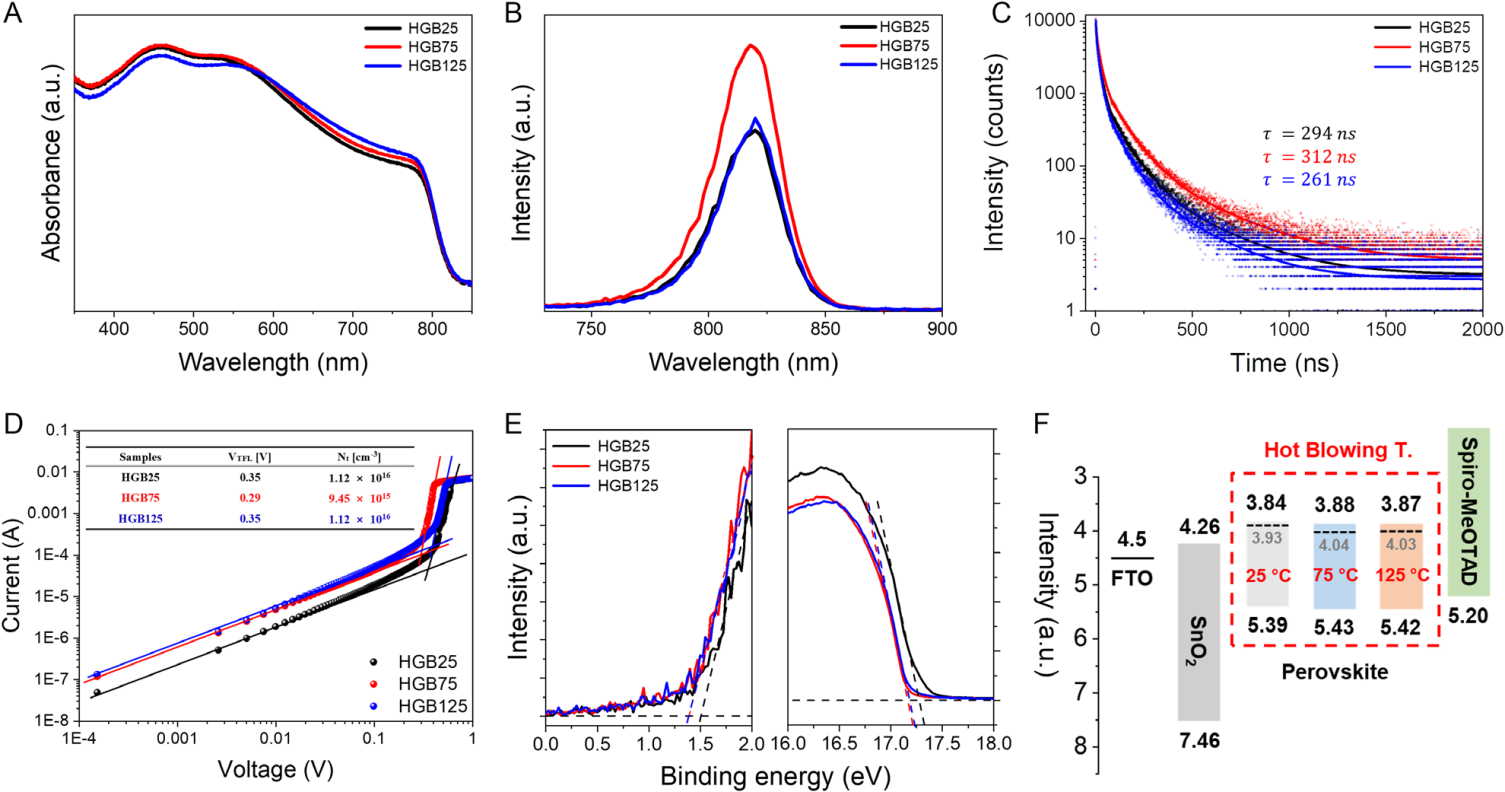
Investigation of the gas‐blowing temperature dependent perovskite films A) UV‐vis absorbance of perovskite films of different gas‐blowing temperatures. (HGB25, HGB75, and HGB125). B) Steady‐state photoluminescence. C) Time‐resolved photoluminescence of perovskite films of different gas‐blowing temperatures, including their recombination lifetime from fitted curves. D) Space charge limit current (SCLC) measurement of perovskites using ETL (electron‐transporting layer)‐based structures; FTO/SnO_2_/perovskite/C_60_/BCP/Ag and calculated values in inset table (trap‐filled limit voltage and trap density). E) Ultraviolet photoelectron spectroscopy (UPS) measurement shows (left) valance band maximum and (right) cut‐off binding energy of perovskite films of different gas‐blowing temperatures. F) Band alignments of perovskite films of different gas‐blowing temperatures derived from UPS data.

To analyze the optical characteristics of perovskite films considering the process temperature, the ultraviolet‐visible (UV‐Vis) absorption and PL spectra were measured. As depicted in **Figure** **3**A, the bandgap extracted from the optical absorption edges of all films shows negligible changes regardless of the process temperature. However, the absorption corresponding to 550 to 800 nm increases as the process temperature increases, though, at the process temperature of the HGB125 condition, it is noted that the absorption signal corresponding to 300 to 500 nm decreases. The change can be interpreted as a decrease in the intensity due to the formation of voids and pinholes, as reported in previous papers.^[^ ^53^, ^54^ ^]^ Figure 3B exhibits the steady‐state PL intensities of the films fabricated at different process temperatures. The perovskite film from the HGB75 condition shows the strongest PL emission compared to the other conditions. Considering that the thicknesses of the perovskite films are similar in Figure S6, Supporting Information, this result indicates that the perovskite film with high crystallinity in HGB75 condition has the lowest nonradiative recombination centers. Defects located on the perovskite surface or at grain boundary can be associated with deep‐level defects where nonradiative recombination losses occur, ultimately decreasing the PL intensity.^[^ ^52^, ^55^ ^]^ The increase in the PL intensity in HGB75 compared to HGB 25 can be interpreted as a decrease of the deep‐level defect density owing to both the improved crystallinity and the lower number of grain boundaries, while the reduction of the PL intensity in the HGB125 condition is due to the formation of voids and pinholes. Figure 3C shows the charge carrier lifetimes of perovskite films created under the different conditions. The carrier lifetimes of the perovskite films from the HGB25, HGB75, and HGB125 conditions are estimated to be 294, 312, and 264 ns, respectively. The improved carrier lifetimes in the HGB75 condition are closely related to the previous PL results. This is also consistent with the increased α‐phase signal in the XRD results in Figure S7, Supporting Information. In relation to this, we calculated the full width at half maximum (FWHM) for the perovskite peaks. The intensity of the (110) diffraction peak became strong with increased gas temperature, and its FWHM significantly decreased from 0.157° (HGB25) to 0.095° (HGB75) and in HGB125 sample showed very similar values with 0.097. This result shows that the crystal quality of the perovskite depends on the gas‐blowing temperature and enlarged grain size. Detailed data are given in the below table. Both steady‐state and time‐resolved PL measurements verify that the perovskite film from the HGB75 condition efficiently suppressed nonradiative recombination paths with relatively fewer deep‐level defects, resulting in an enhanced carrier lifetime.

Space‐charge limited current (SCLC) measurements were also performed to investigate the electron trap density of the perovskite film according to the temperature of the blowing gas.^[^ ^56^, ^57^ ^]^ Electron‐only types of devices were prepared with the structure of FTO/SnO_2_/perovskite/C_60_/BCP/Au. As shown in Figure 3D, an ohmic region where the bias voltage has a linear relationship with the current density voltage was observed. The trap‐filled limit voltage (*V*_TFL_) can be derived to confirm the intersection of the voltage between the ohmic region and the trap‐filled region. *V*_TFL_ outcomes of the perovskite film fabricated from the HGB25, HGB75, and HGB125 conditions are 0.35, 0.29, and 0.39 V, respectively. The trap density (*N*_t_) can be determined from the following equation^[^ ^56^, ^57^ ^]^

(4) $$V_{\text{TFL}}\;=\;\frac{eN_{t}d^{2}}{2\varepsilon \varepsilon_{0}}$$

where *e*, *d*, *ε*, and *ε*_0_ are the elementary charge, the thickness, the vacuum permittivity, and the relative dielectric constant of the perovskite films, respectively. The relative dielectric constant of perovskite is assumed to be 46.9.^[^ ^58^ ^]^ Thickness of the perovskite film is about 400 nm that can be derived from the cross‐sectional SEM image as shown in Figure S6, Supporting Information. The calculated N_t_ values of the perovskite film in the HGB25, HGB75 and HGB125 cases are 1.121 × 10^16^, 9.450 × 10^15^, and 1.124 × 10^16^ cm^−3^, respectively. (see Figure 3D, inset table) This result indicates that the perovskite film from the HGB75 condition shows a significant decrease in the trap density and is in good agreement with previous FLIM and PL outcomes.

To identify the effect of the blowing gas temperature on the electronic structure of the perovskite film, ultraviolet photoelectron spectroscopy (UPS) measurements were taken. Figure 3E and Table S2, Supporting Information, show valance band maximum and cutoff binding energy of perovskite films with different gas‐blowing temperatures. As shown in Figure 3E, the values of the energy difference from the valence band to the Fermi level and the Fermi level in film under the HGB25 condition are 1.46 and 3.93 eV. On the other hand, for the HGB75 and HGB125 perovskite films, the energy difference from the valence band to the Fermi level in film is 1.39 eV, lower than the value for the HGB25. Both the HGB75 and HGB125 films show similar energy alignment of the *E*_F_ at 4.03 and 4.02 eV, respectively. These results indicate that the HGB25 film processes relatively stronger n‐type properties than the HGB75 and HGB125 films considering the similar bandgap of perovskite films. Figure 3F shows a schematic illustration that expresses the energy alignment of perovskite films created at different temperatures based on UPS measurements. We confirmed the change in the type in the perovskite film depending on the blowing gas temperature. The change in the type of perovskite thin film is determined by the type of defect, known as the self‐doping effect.^[^ ^59^, ^60^ ^]^ When many n‐type defects exist in a thin film, a perovskite thin film exhibits n‐type properties. On the other hand, if there are many p‐type defects inside the thin film, a perovskite thin film has p‐type properties. The types of defects in the perovskite film can be adjusted in the atomic ratio between lead (Pb) and iodine (I).^[^ ^59^, ^61^ ^]^ Previous studies have reported that perovskite films with Pb‐rich conditions and Pb‐deficient conditions exhibited n‐type and p‐type properties, respectively, since the defect formation energy of the defects relies on the atomic ratio between Pb and I.^[^ ^62^, ^63^ ^]^ To gain insight into the change of WF, the atomic ratio of the iodide elements with respect to Pb is compared with respect to the blowing gas temperature as shown in Table S3, Supporting Information. The I to Pb ratio of the HGB25 perovskite film is 2.76, while the I to Pb ratio of HGB75 and HGB125 perovskite films is 2.84 and 2.86, respectively. The I/Pb ratio increases as the process temperature increases, implying that process temperature plays a critical role in the overall perovskite composition. We also found that a high blowing gas temperature facilitates the fabricating perovskite film that approximates an intrinsic semiconductor and a complete perovskite composition compared to the perovskite film fabricated using gas at room temperature. This result arises because blowing gas at a high temperature imparts in the perovskite film a low defect concentration and facilitates fast evaporation of the solvent, showing an effect similar to that of hot casting.^[^ ^27^, ^29^ ^]^

**Figure 4:**
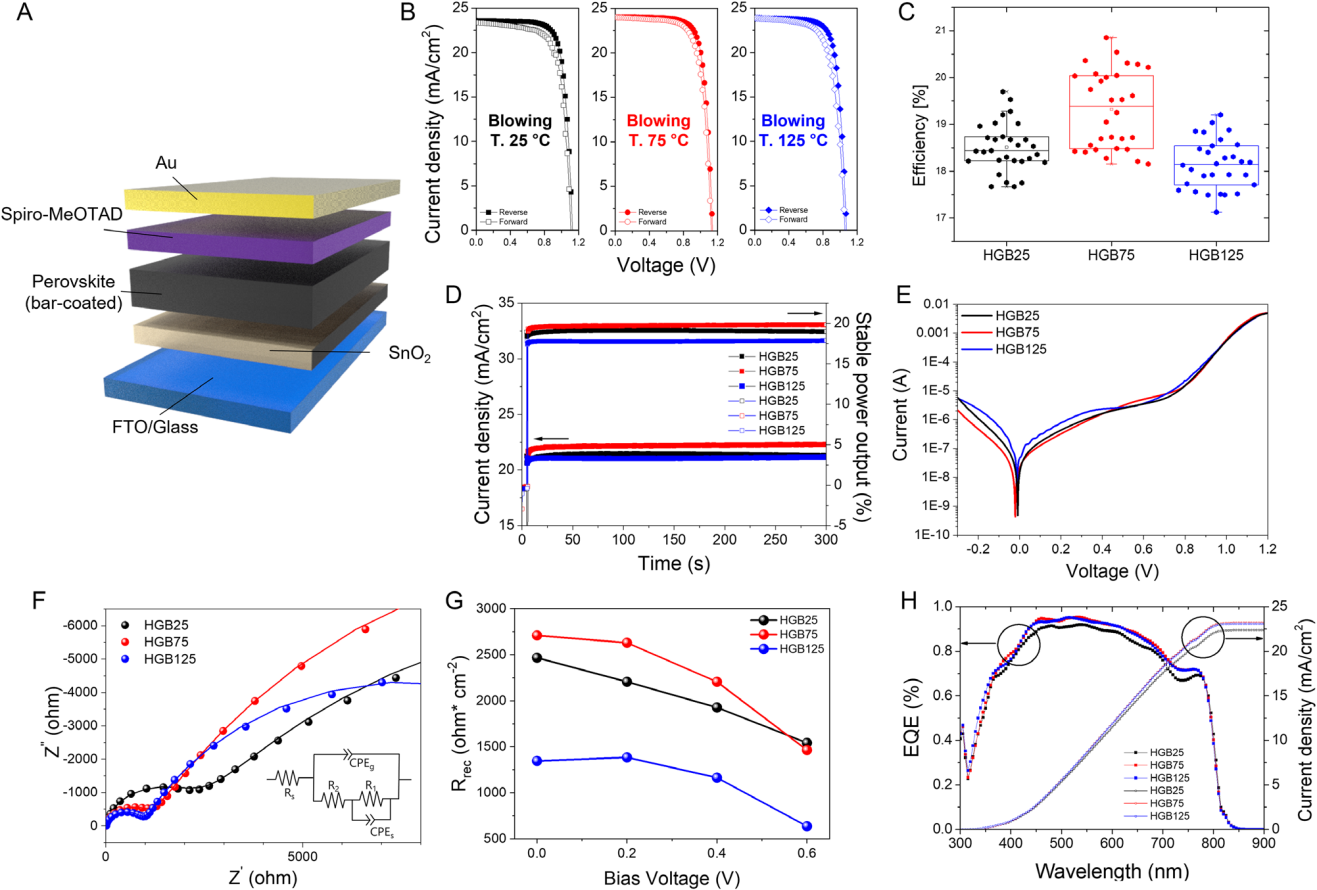
Schematic and characteristics of bar‐coated perovskite solar cells. A) Scheme of structure of a perovskite solar cell; FTO/SnO_2_/perovskite/Spiro‐OMeTAD/Au. B) *J–V* measurements of perovskite solar cells fabricated by using different gas‐blowing temperatures; (left) HGB25, (middle) HGB75, and (right) HGB125, respectively. C) Power conversion efficiency (PCE) statistics perovskite solar cells of different gas‐blowing temperatures. D) Maximum power point tracking (MPPT) and stable power output (SPO) measurements of perovskite solar cells with different gas‐blowing temperatures. E) dark current measurements of perovskite solar cells of different gas‐blowing temperatures. F) Electrochemical impedance spectroscopy (EIS) of perovskite solar cells of different gas‐blowing temperatures: Nyquist plots (the inset figure shows the equivalent circuit for the calculation) G) Recombination resistance was calculated from the Nyquist plots. H) External quantum efficiency (EQE) and their calculated current density (*J*_sc_) of perovskite solar cells of different gas‐blowing temperatures.

The performance of PSCs depending on the blowing gas temperature is evaluated. The structure of PSCs can be summarized as FTO/SnO_2_/Cs‐doped (FA_*x*_MA_*y*_)Pb(I_*z*_Br_1−*z*_)/Spiro‐OMeTAD/Au as illustrated in **Figure** **4**A. Figure 4B shows the *J–V* curves of the PSCs showing the best performance from both reverse and forward scans, with detailed data described in Table S4, Supporting Information. More than 30 samples PCE were listed and sorted. The highest PCEs at a reverse scan for the HGB25, HGB75, and HGB125 PSCs are 19.70%, 20.85%, and 19.20%, respectively (see Table S5, Supporting Information). While the PSCs for HGB25 and HGB75 show smaller *J–V* hysteresis (HI 3.55% for HGB25 and HI 3.27% for HGB75), the PSC for HGB125 shows noticeable *J–V* hysteresis (HI 5.38% by HGB125). This result is possibly due to considerable roughness and the numerous voids in the perovskite film of HGB125. The existence of roughness and many voids facilitate charge accumulation and ion migration at the interface, aggravating the *J–V* hysteresis. Figure 4C shows statistical histograms of the *J–V* characteristics of the fabricated PSCs under each condition. The average PCEs of the PSCs by the HGB25, HGB75, and HGB125 conditions were measured to be 18.51%, 19.32%, and 18.14%, respectively. Among them, the PSCs from the HGB75 condition exhibited the highest PCE with increased open‐circuit voltage (*V*_OC_), short‐circuit current density (*J*_sc_), and fill factor (FF) compared to PSCs of HGB25 and HGB125. Figure S8, Supporting Information, shows the statistics of photovoltaic parameters of *V*_OC_, *J*_SC_, and FF for the HGB25, HGB75, and HGB125 samples based on the data from 30 cells for each case. These results are consistent with previous results of PL mappings and SCLC. As shown in Figure 4D, the stabilized power output (SPO) of the PSCs at different temperatures shows a trend similar to that of the PCE measured from *J–V* scan. PSCs from the HGB75 condition exhibited 20.0% at a maximum power point voltage of 0.89 V, showing a negligible PCE difference from the measured *J–V* scan. Additionally, we further examined the long‐term photostability for encapsulated HGB75 optimized condition sample under 1 sun (RT, 100 mW cm^−2^) without a cooling system, and a UV cut filter reveals that the device shows reliable photostable PSCs and maintains about 93% of initial PCE after 300 h. (see Figure S9, Supporting Information) In this measurement, we applied a voltage of 0.7 V, which is approximately 90% of MPPV (voltage at maximum power point), due to concerns about deterioration of the solar cell during the encapsulation process in an ambient air and the system not being able to track performance in real time. To evaluate the diode quality, the dark current density–voltages of PSCs with different process temperatures were scanned. The dark saturation current can be extracted from the dark current density–voltage curve on semi‐logarithmic plots. The dark saturation current (*J*_0_) is closely related to the quality of a perovskite solar cell. Increases of *J*_0_ indicate poor diode properties and an increase in the charge carrier recombination.^[^ ^64^, ^65^ ^]^ As depicted in Figure 4E, the PSC from the HGB75 condition shows a low dark saturation level, indicating good diode behavior with a relatively high open‐circuit voltage. Additionally, electrochemical impedance spectroscopy (EIS) was explored to elucidate the working mechanisms of the PSCs fabricated with different applying temperatures. Figure 4F,G shows Nyquist plots with an inset equivalent circuit for the fitting and recombination resistance calculated from the Nyquist plots, respectively.^[^ ^66^ ^]^ The recombination resistance of the PSCs from the HGB75 condition shows a higher value than that from the HGB25, implying that the PSC from the HGB75 condition effectively suppressed recombination losses. Specifically, the recombination resistance of the PSCs by HGB125 shows the lowest value given that the many voids and pinholes existing in this case can hinder efficient charge transfers. The EIS results indicate that the lowest recombination losses of PSCs from the HGB75 condition result in an increase of *V*_OC_ and FF. To investigate the incident‐photon‐to‐electron conversion efficiency, we acquired external quantum efficiency (EQE) spectra with a wavelength range between 300 and 900 nm (see Figure 4H). The integrated *J*_SC_ of PSCs calculated from the external quantum efficiency (EQE) values from the HGB25, HGB75, and HGB125 cases are correspondingly 22.4, 23.2 and 23.1 mA cm^2^, similar to the measured *J*_SC_ value and integrated value of the PSCs under 1 sun illumination (see Figure S8 and Table S5, Supporting Information). Compared to current density of the HGB25 and HGB125 PSCs, the HGB75 PSCs achieved higher integrated current densities, indicating efficient charge extraction owing to their improved film quality levels and low shunt paths with uniform coverage, as mentioned in the discussion of our previous results.

**Figure 5:**
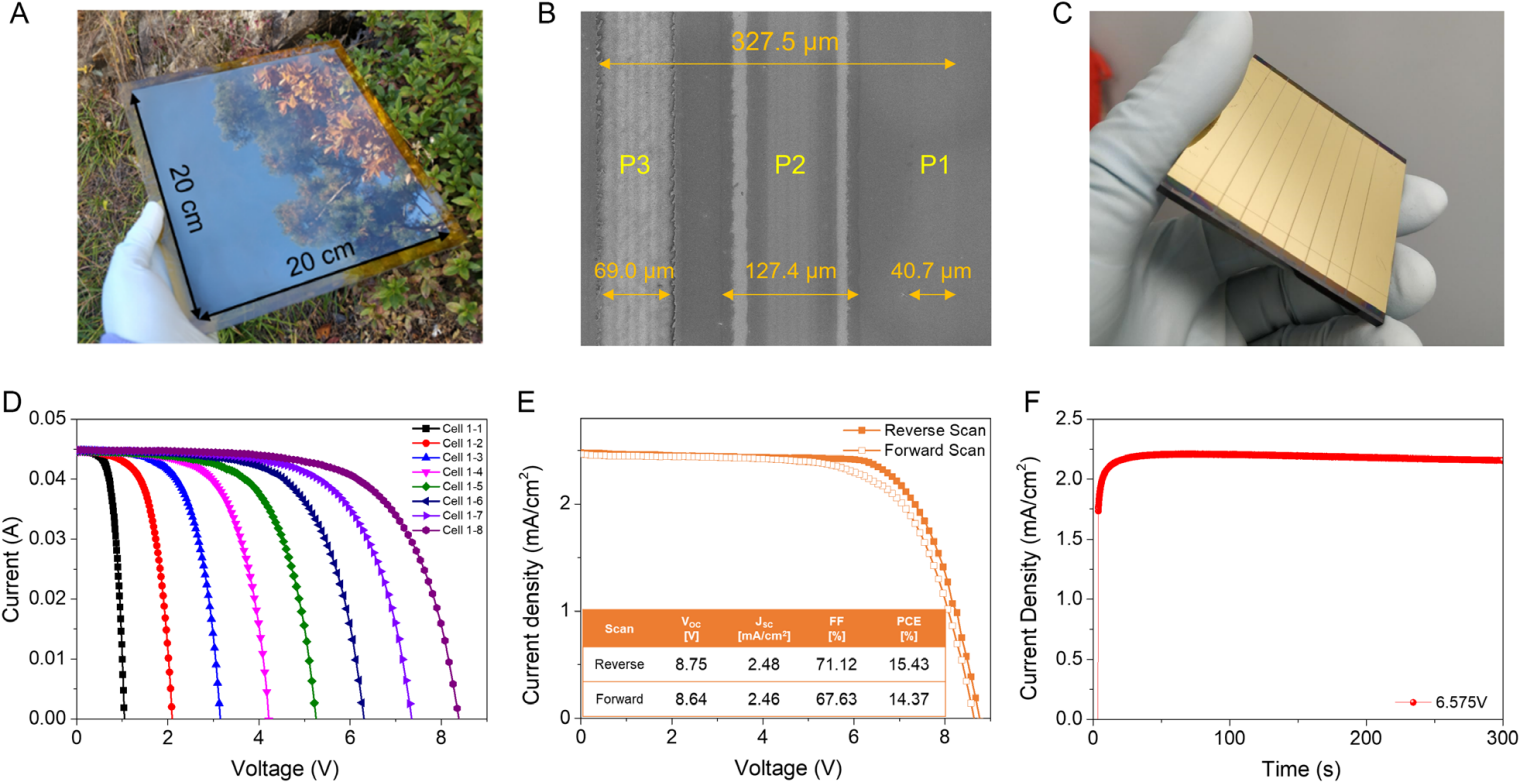
Images of laser‐patterned perovskite module and characteristics. A) Perovskite film deposited on 400 cm^2^ substrate fabricated by hot gas‐blowing‐assisted bar coating. B) SEM images of the line widths of laser‐patterned P1, P2, and P3. GFF factor is about 93.45% C) Photograph of the laser‐patterned perovskite module. D) *J–V* curves for each cell connected in series in a perovskite module. The module was separated by connecting 8 cells, and the measurement was characterized by increasing the number of connected cells. E) *J–V* characteristics of the best performance perovskite module. Active area is 18 cm^2^. F) Steady‐state current measurement of the laser‐patterned module at a maximum voltage.

We evaluated the compatibility of the hot gas‐blowing technique with upscaling processes by fabricating perovskite modules by means of the bar‐coating method. To check whether a uniform large‐area coating is possible, a perovskite film of an area of close to 400 cm^2^ is fabricated from the HGB75 condition on a glass substrate. As shown in **Figure** **5**A and S10, Supporting Information, the perovskite film with a clean surface can be found. In addition, ellipsometry analysis is conducted to measure the actual thickness distribution of the perovskite film. The perovskite layer is formed by the same coating method on a Si wafer with an area of 100 cm^2^ (see Figure S11, Supporting Information). A uniform distribution of the thickness is confirmed, and the average value of thickness of the perovskite layer is 391.06 nm, which shows a similar result as the value obtained in the SEM cross‐section image in Figure S6, Supporting Information. Perovskite solar modules are fabricated with an aperture area of 18 cm^2^ (Figure 5C) The modulation is performed by three times of laser etching process (P1, P2, and P3), and the geometric fill factor (GFF) of the best performed modules was 93.45% (see Figure 5B and S12, Supporting Information). The large size module with eight subcells showed high aperture efficiencies of 13.78% (*V*_OC_ = 1.04 V, *J*_SC_ = 19.92 mA cm^−2^, and FF = 0.613% for each subcell with an area of 2.25 cm^2^, as shown in Figure 5D). A best‐performing module shows a PCE of 15.43% with *V*_OC_ = 8.75 V, *J*_SC_ = 2.48 mA cm^−2^, and FF = 0.711, and the perovskite module maintained its performance well from MPPT measurement (see Figure 5E,F). As a result, it is shown that our hot gas‐blowing method for PSCs is consistently applied even in the large‐area production.

## 3 Conclusion

We have developed a large‐area bar coating method that adopted hot gas‐blowing technique that allows for grain size controllable high crystalline PSCs. In large‐area process, the selection of the solvent and evaporation rate and grain growth mechanism are crucial factors to acquire high crystalline perovskite film. Developed HGB method at an appropriate blowing temperature affected both high volatile 2ME solvent‐based perovskite precursor evaporation control and the grain growth mechanism, which could make an optimal condition for perovskite crystallization. Compared to HGB25 and HGB125, HGB75 produced void‐free and planar perovskite layer with large grain size, resulting in improvement of optical and electrical properties such as carrier lifetime and low trap density. The HGB75 perovskite solar cell showed the highest PCE of 20.85% among all gas‐blowing temperature conditions and high photostability over 300 h. The 25 cm^2^ sized perovskite solar modules of the HGB75 showed PCE of 15.4%, indicating feasibility with large area and stable fabrication.

## 4 Experimental Section

4.1

4.1.1

##### Materials

Substrates (FTO/glass and bare glass) were purchased from AMG Tech. Tin(IV) oxide (SnO_2_) colloidal dispersion (15% in H_2_O) and Pb‐related materials (PbI_2_ and PbCl_2_) were purchased from Alfa Aesar. CsI was purchased from TCI Chemical. Ammonium halides (FAI, PEABr) were purchased from Greatcell Solar Ltd., 2,2′,7,7′‐tetrakis(*N*,*N*‐di‐*p*‐methoxyphenylamino)‐9,9′‐spirobifluorene (Spiro‐OMeTAD) was purchased from Lumtec. Au pellets for top electrode were purchased from iTASCO. All other materials were purchased from Sigma Aldrich.

##### Solar Cell Fabrication

FTO glass was cleaned by sonication for 15 min with acetone, 2‐propanol, and DI water. SnO_2_ solution for device fabrication was made by mixing the SnO_2_ colloidal solution and DI water in a volume ratio 1:4. The SnO_2_ solution was spin coated on the FTO glass with 4000 rpm for 30 s and annealed at 150 °C for 30 min. Perovskite precursor solution was made to a concentration of 1.5 m by mixing following materials in 2‐methoxyethanol (2ME); 461 mg of PbI_2_, 13.9 mg of PbCl_2_, 206.4 mg of FAI, 7.2 mg of CsI, 1.3 mg of PEABr, and 100 μL of 1‐methyl‐2‐pyrrolidinone (NMP) in 0.8 mL of 2ME. The substrate was loaded on a bar‐coating equipment, and 100 μL of the precursor solution was dropped on the substrate and filled a gap between the substrate and the bar. The perovskite layer was fabricated by the bar moving at a speed of 40 mm s^−1^, followed by blown by an air knife with nitrogen gas with a pressure of 20 psi followed at the same speed, and annealed at 150 °C for 10 min and 100 °C for 10 min. The gaps between the substrate and the bar and between the substrate and the air knife are 100 and 500 μm, respectively. Spiro‐OMeTAD solution was prepared by dissolving 90 mg of the Spiro‐OMeTAD in 1 mL of chlorobenzene (CB) with addition of 39.5 μL of tBP, 23 μL of Li‐TFSI solution (520 mg of bis(trifluoromethane)sulfonimide lithium salt in 1 mL of acetonitrile), and 10 μL of FK209 Co(III)‐TFSI solution (375 mg of the tris(2‐(1H‐pyrazol‐1‐yl)‐4‐tert‐butylpyridine)‐cobalt(III)tris(bis(trifluoromethylsulfonyl)imide)) in 1 mL of acetonitrile). The Spiro‐OMeTAD solution was spin coated on the perovskite layer with 3000 rpm for 30 s. Finally, a 50 nm thickness of Au electrode was fabricated by thermal evaporation.

##### Large Area Solar Module Fabrication

Modulation for large area solar device was conducted via picosecond laser scriber (wavelength of the laser is 532 nm). Three laser scribing (P1, P2, and P3) were performed: P1 was for separation of the FTO electrode, P2 was for interconnection of top and bottom electrodes, and P3 was for separation of the Au electrode. The other procedures for fabrication of each layer were the same as for solar cell fabrication.

##### Characterization

SEM images of top view and cross‐section view of perovskite layers were obtained from field emission scanning electron microscope (Merlin Compact; ZEISS) with 2 kV working voltage. AFM, C‐AFM images, and roughness data of perovskite layers were obtained from AFM (NX10; Park systems). Steady‐state PL was conducted via steady‐state and lifetime spectro fluorometers (Fluoromax‐4; Horiba). Time‐resolved PL and PL mapping were conducted by time‐resolved fluorescence confocal microscope (Microtime‐200; PicoQuant). Absorbance of perovskite layers was measured via by using UV‐Vis‐NIR Spectrometer (Cary 5000; Agilent). XPS and UPS analyses were conducted by electron spectroscopy for chemical analysis (AXIS SUPRA; Kratos). SCLC and EIS measurements were performed by using electrochemical workstation (AUT302N; Metrohm Autolab). *J*–*V* measurement and maximum power point tracking (MPPT) of PSCs were conducted via a sourcemeter (Keithley2400; Tektronix) and a solar simulator from Newport (94023A; Newport) with the reference cell (91 150‐KG3; Newport) for calibration. EQE data of PSCs were measured from quantum efficiency measurement solution (IQE200B‐E; Newport). XRD data of perovskite layers were obtained from an X‐ray diffractometer (D8 advance, Bruker) in a range of 2*θ* of 5–80°.

## Conflict of Interest

The authors declare no conflict of interest.

## Supporting information

Supplementary Material

## Data Availability

The data that support the findings of this study are available from the corresponding author upon reasonable request.
