# Supplementary material for: Hot Gas‐Blowing Assisted Crystallinity Management of Bar‐Coated Perovskite Solar Cells and Modules
Source: Small Sci. 2023 Jul 18;3(9):2300069. doi: 10.1002/smsc.202300069 (PMC11935980; doi:10.1002/smsc.202300069)
Supplement: Supplementary file 1 — Supplementary Material [file SMSC-3-2300069-s001.pdf]

Supporting Information

Hot Gas-Blowing Assisted Crystallinity Management of Bar-Coated Perovskite Solar Cells and Modules

Dr. M. Han, Dr. J. Byeon, C. Hur, Prof. M. Choi
Department of Mechanical Engineering, Seoul National University, Seoul 08826, Republic of Korea
Global Frontier Center for Multiscale Energy Systems, Seoul National University, Seoul 08826, Republic of Korea
E-mail: mchoi@snu.ac.kr

J. Jang, Dr. G. Seo and Prof. M. Choi
Frontier Energy Solution Corporation, Seoul National University, Seoul 08826, Republic of Korea
E-mail: photoacoustic.image@gmail.com, mchoi@snu.ac.kr

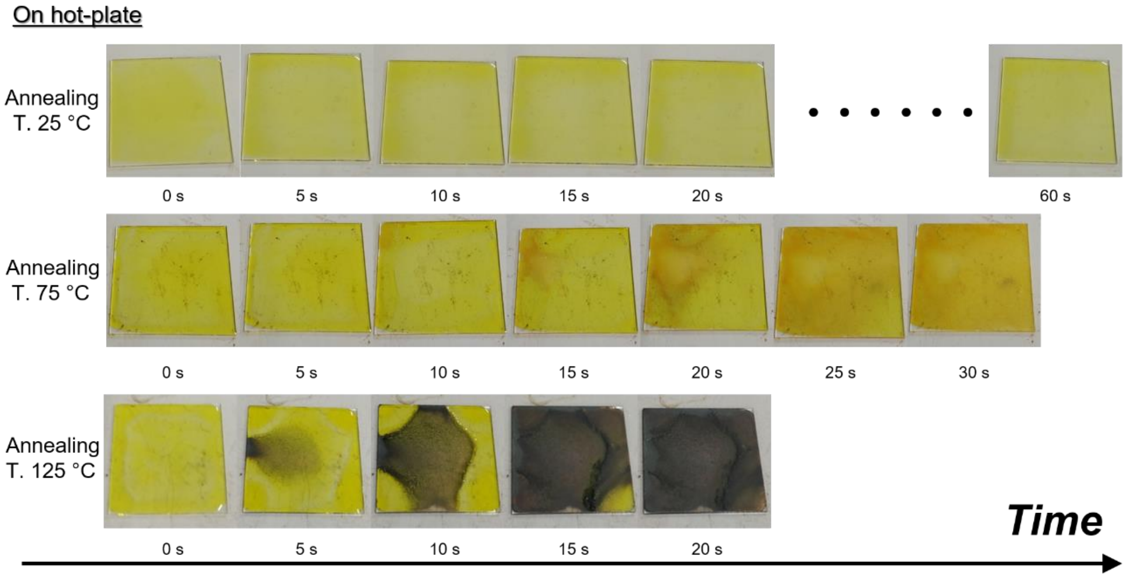

Fig. S1. Photographs of change of perovskite precursor solution on different temperatures of hot plate up to 60 sec. The solution at room temperature 25 ℃ (upper line) keeps its original status, however, the solution at 75 ℃ (middle line) or 125 ℃ (bottom line) is changed to non-homogeneous status quickly. (within 20-30 sec).

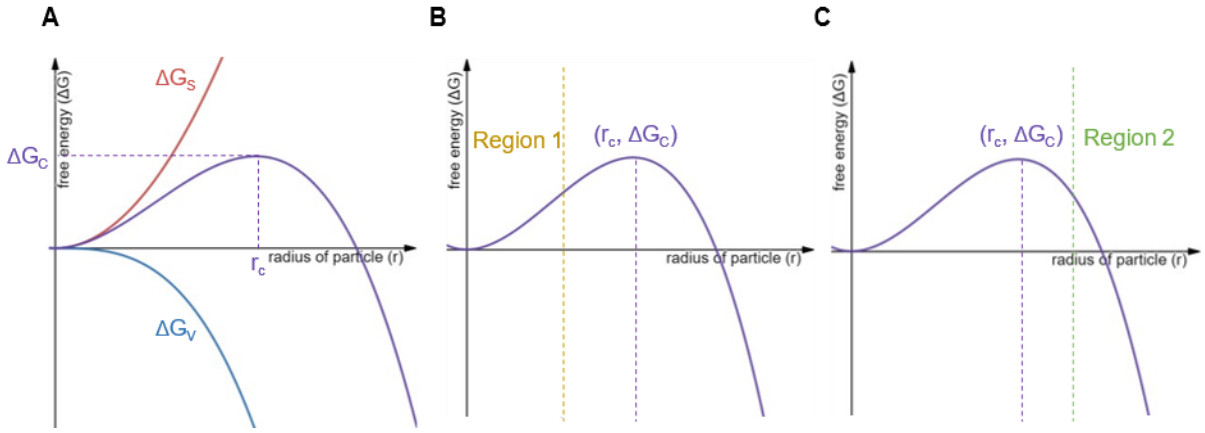

Fig. S2. a) Schematic illustration of free energy diagram for nucleation showing critical radius of nucleus and critical gibbs energy barrier. b). Schematic illustration of crystallization process in HGB25. The nucleus must overcome the gibbs energy barreir to continuously grow (Region 1). c) Schematic illustration of crystallization process in HGB75 and HGB125. the crystallization initiated spontaneously due to larger radius of nucleus than critical radius in HGB75 and HGB125 (Region 2)

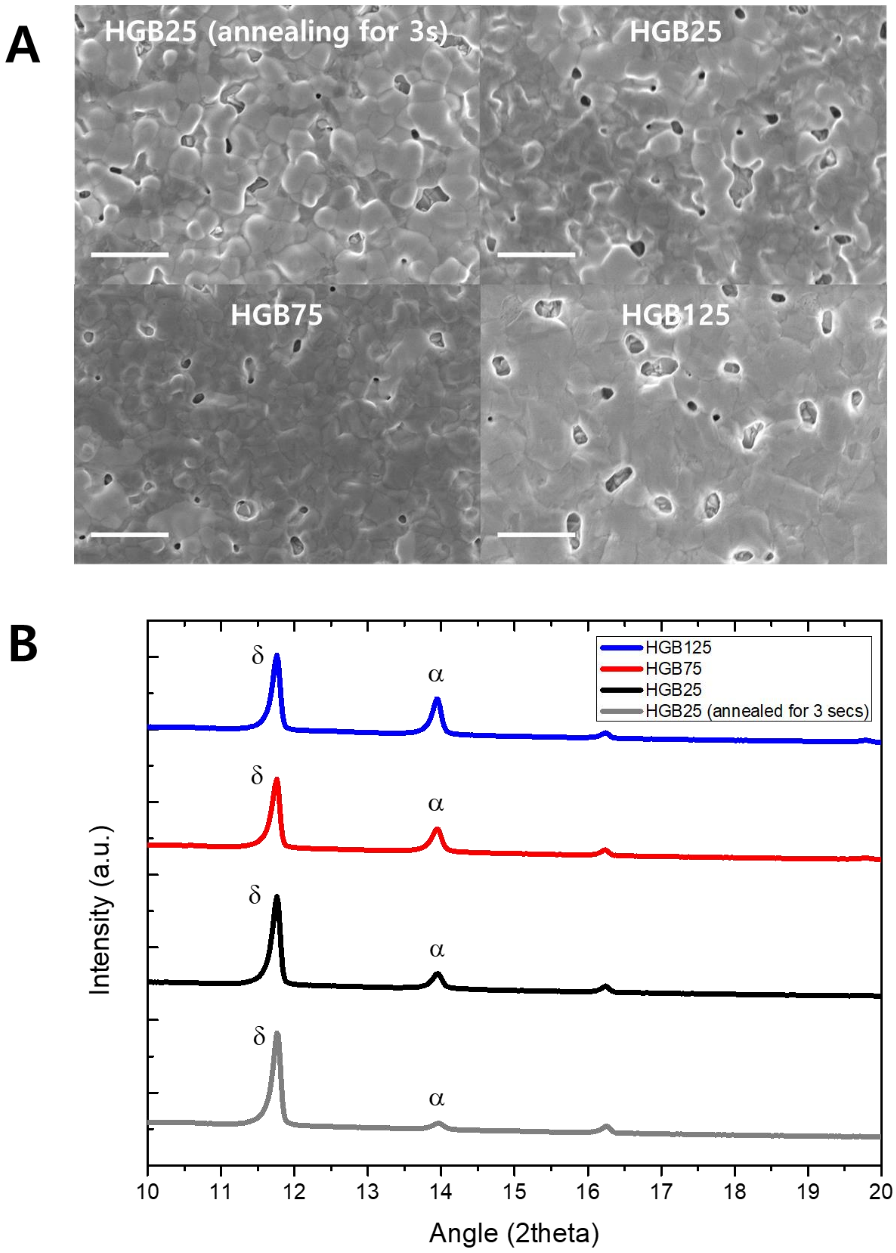

Fig. S3. a) SEM images and b) XRD measurements of perovskite films annealed for short times (3 secs or 7 secs) on the 150 °C hot plate after gas blowing with different temperatures. The scale bar of the SEM image is 1 μm. For the XRD data, the alpha phase and the delta phase of the perovskite are represented at 2θ = 11.7º and 13.9º, respectively.

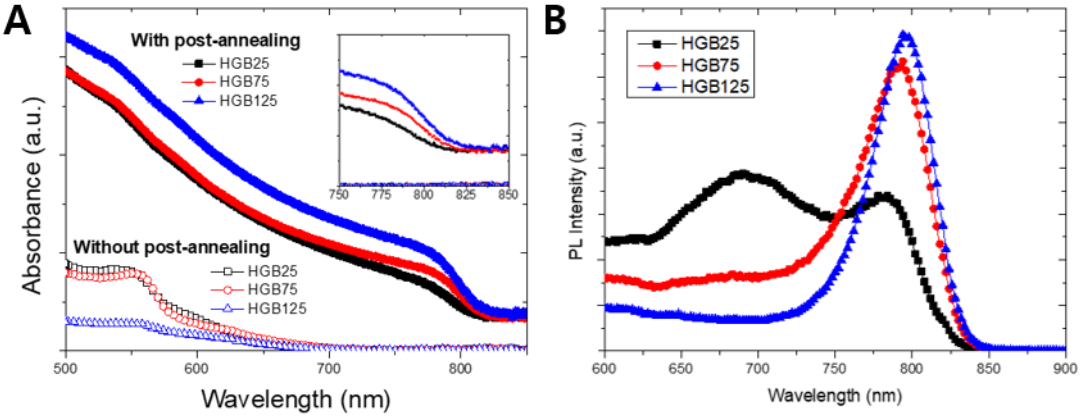

Fig. S4. a) UV-vis absorbance measurement of perovskite films of HGB25, HGB75, and HGB125 with short annealing for 7 seconds on the 150 °C hot plate (filled symbols) and without post-annealing on a hot plate (open symbols). An inset in the UV-vis absorbance is the same data as in the Fig. S4a with the wavelength range set from 750 nm to 850 nm. b) Steady-state photoluminescence of perovskite films of HGB25, HGB75, and HGB125 with short annealing for 7 seconds on the 150 °C hot plate.

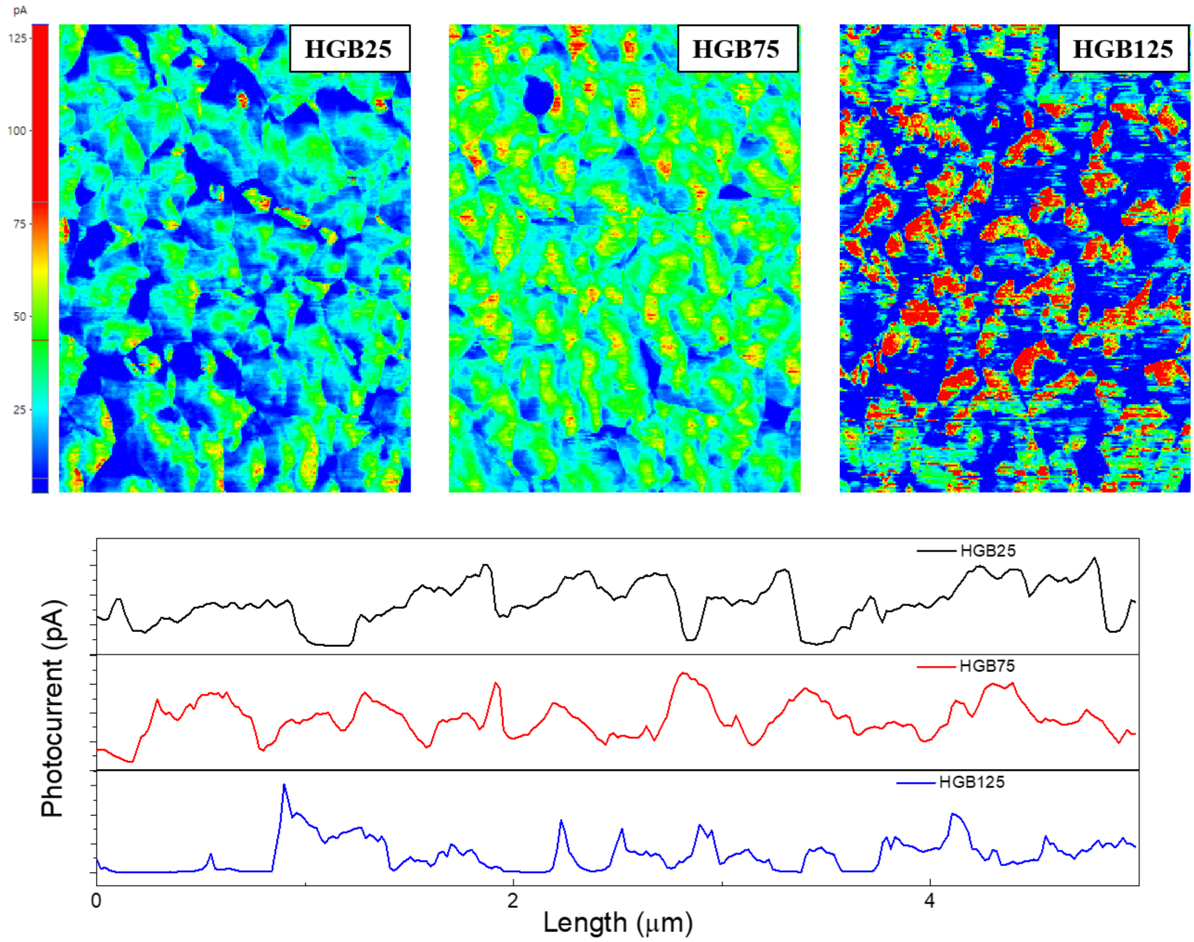

Fig. S5. C-AFM images of the perovskite films of different gas blowing temperatures. The current distribution of perovskite is strongly dependent on surface roughness. Thus, HGB25 and HGB75 c-AFM overall current intensities are homogeneous, while HGB125 surface current intensity is incoherent. The difference in surface roughness height is large and the surface current according to the c-AFM measurement result is also a big difference.

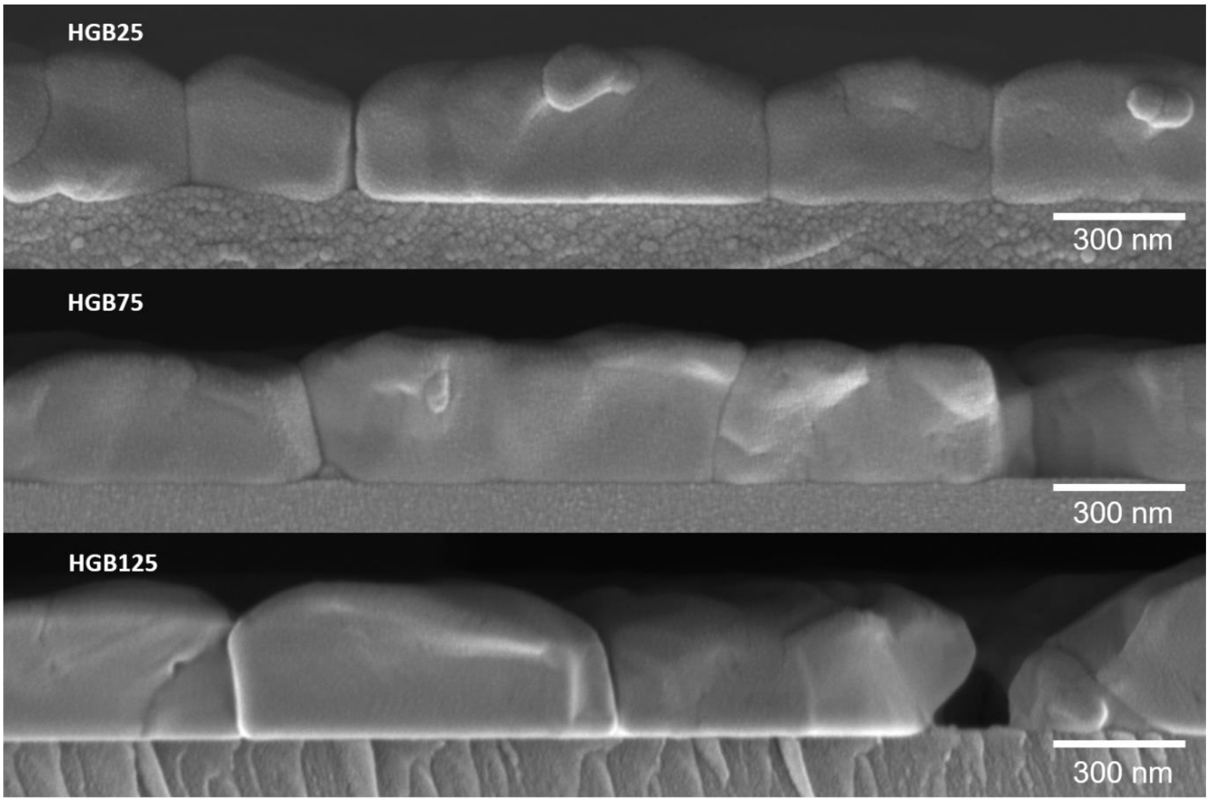

Fig. S6. Cross-sectional SEM images of perovskite layers of different gas-blowing temperatures. (HGB25, HGB75, and HGB125) on the glass substrate. The perovskite layers of all conditions show a similar thickness of about 400 nm and good crystallinity in a vertical direction. In HGB25, perovskite grains are close-packed but their size is small. In HGB75, large perovskite grains are densely formed. In HGB125, large grains are formed but voids are present.

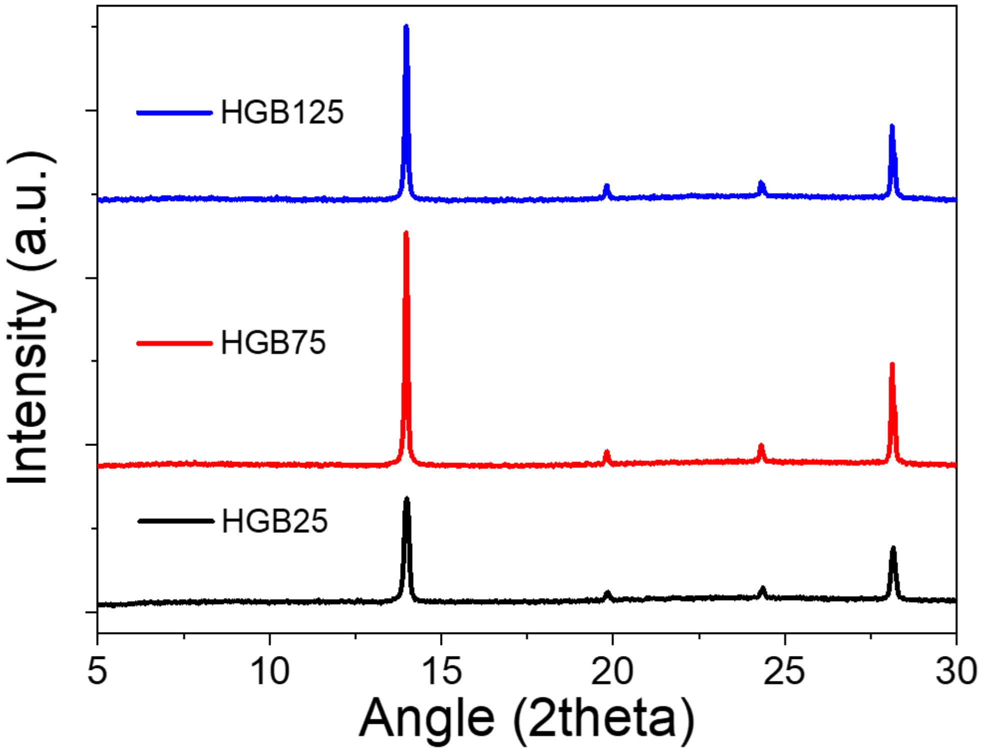

Sample	HGB25	HGB75	HGB125
FWHM	0.157	0.095	0.097

Fig. S7. XRD graph of different gas blowing temperature and their full-width at half maximum (FWHM) data. Under all conditions, the alpha phase of the perovskite is confirmed without any signal of PbI_2 or delta phase of the perovskite. The FWHM data were calculated from peaks representing the alpha phase of the perovskite.

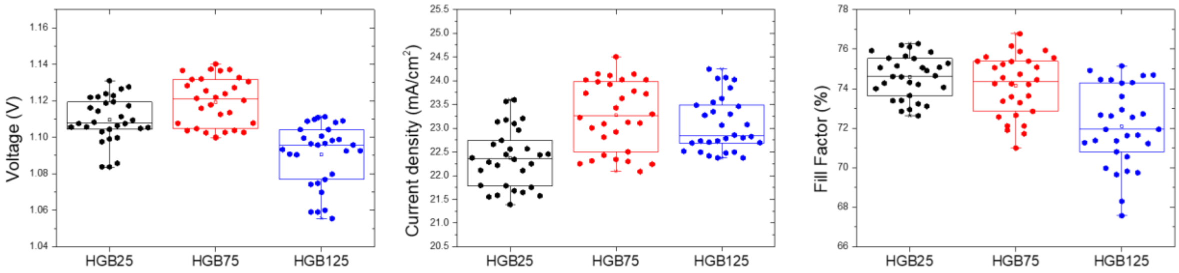

Fig. S8. Statistical data of perovskite solar cells of HGB25, HGB75, and HGB 125; (left) V_{OC}, (middle) J_{SC}, and (right) fill factor.

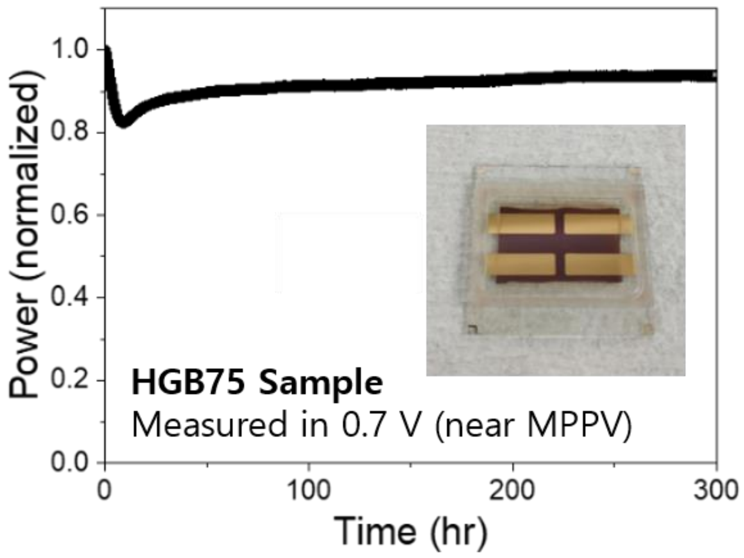

Fig. S9. Long-term photostability measurement of the HGB75 sample. The device was encapsulated and the measurement was conducted under 1 sun illumination and near maximum power point voltage at room temperature. To measure the long-term stability of the perovskite solar cell in this system, we applied glass to glass encapsulation to the solar cell sample, and ITO-ITO electrodes were connected with a self-made station, instead of using a gold probe. The photograph of the encapsulated solar cell is placed in the figure as an inset image. The HGB75 device maintained about 93% of its original power after 300 hours.

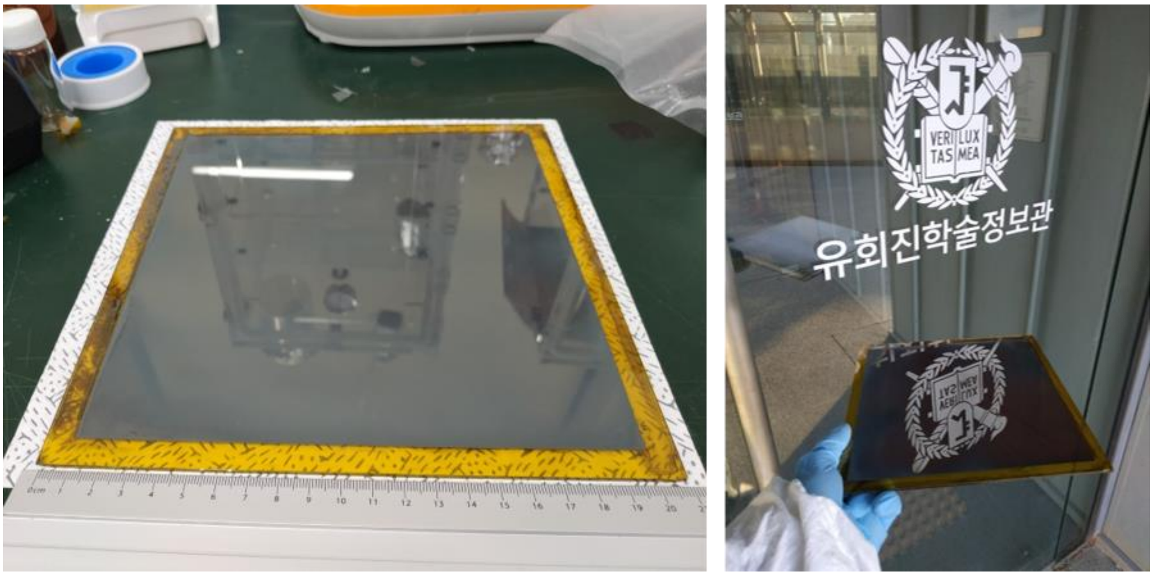

Fig. S10. Detailed photos of the bar coated perovskite film on an area of 400 cm^2. The substrate is a square with a side length of about 20 cm (left) and the perovskite film shows a glassy surface. (right)

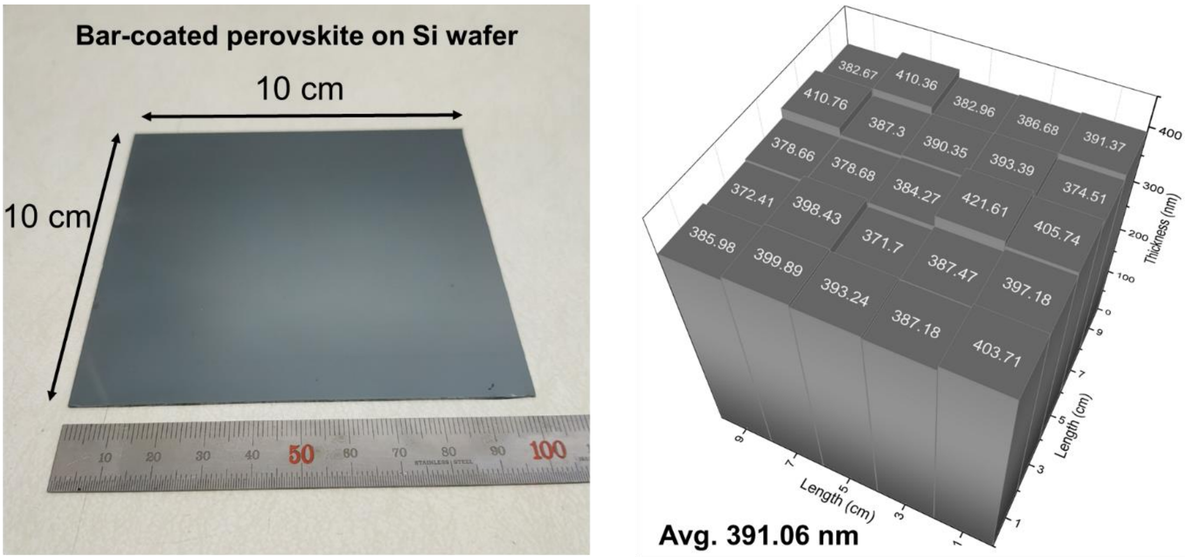

Fig. S11. The thickness of bar coated perovskite layer via ellipsometry Photograph of bar-coated perovskite layer on Si substrate (left) and measured thickness (right). The average thickness of total 25 points is 391.06 nm.

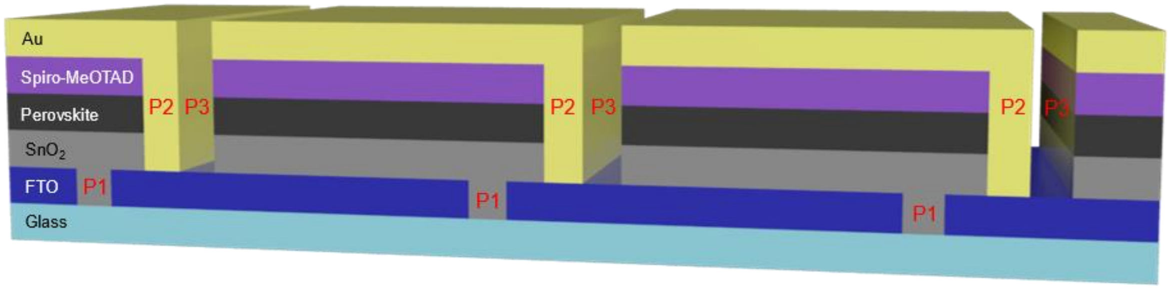

Fig. S12. Configuration of perovskite solar modules with modulation by laser etching process (P1, P2, and P3). Structure of the solar modules is FTO (Glass)/SnO_2/Perovskite/Spiro-MeOTAD/Au, which is the same structure as fabricated in perovskite solar cells. P1 shorts the FTO electrode, P2 etches from the ETL to the HTL to interconnect sub-cells by Au electrode, and P3 shorts the Au electrode.

Table S1. RMS roughness data of perovskite surfaces of different gas blowing temperatures.

Sample	Rq[a)] (nm)	Ra[b)] (nm)	Rz[c)] (nm)
HGB25	23.31	18.78	157.91
HGB75	21.23	16.77	130.25
HGB125	32.08	25.80	190.08

[a)] Root mean square roughness; [b)] Roughness average; [c)] Average maximum height of the profile

Table S2. Cut-off energy and work function calculated from UPS data of perovskite films of different gas-blowing temperatures. Cut-off energy and valance band maximum was calculated from fitted curves based on He photon source (21.22 eV). Work function is derived from those values.

Conditions	HGB25	HGB75	HGB125
Cut-off (eV)	17.29	17.18	17.19
Work Function (eV)	3.93	4.04	4.03
Valence Band Maximum (eV)	5.39	5.43	5.42

Table S3. XPS data of perovskite layers of HGB25, HGB75, and HGB125 can be checked for the concentration of each constituent element (C, N, O, Pb, I, and Br). The ratio of I to Pb could be calculated from the data, which is associated with the formation of p-type defects.

Quantification								
HGB25	BE [eV]	FWHM [eV]	RSF	Atomic conc. [%]	Error [%]	Mass conc. [%]	Error [%]	I/Pb ratio
Br 3d	71.09	0.00	1.06	0.3	0.10	0.4	0.14	
O 1s	531.59	0.00	0.78	3.4	0.40	0.9	0.11	
N 1s	399.99	0.00	0.48	16.5	0.49	3.9	0.12	
Pb 4f7	137.89	0.00	4.76	9.1	0.15	31.8	0.21	I/Pb 2.76116
I 3d5	618.59	0.00	6.21	25.2	0.37	53.8	0.25	
C 1s	284.49	0.00	0.28	45.4	0.77	9.2	0.24	
HGB75	BE [eV]	FWHM [eV]	RSF	Atomic conc. [%]	Error [%]	Mass conc. [%]	Error [%]	
Br 3d	71.29	0.00	1.06	0.2	0.09	0.3	0.11	
O 1s	530.39	0.00	0.78	2.9	0.40	0.7	0.10	
N 1s	399.99	0.00	0.48	19.4	0.55	4.0	0.12	
Pb 4f7	137.89	0.00	4.76	10.5	0.18	32.3	0.20	I/Pb 2.83697
I 3d5	618.59	0.00	6.21	29.8	0.48	56.1	0.24	
C 1s	284.39	0.00	0.28	37.1	0.92	6.6	0.23	
HGB125	BE [eV]	FWHM [eV]	RSF	Atomic conc. [%]	Error [%]	Mass conc. [%]	Error [%]	
Br 3d	71.29	0.00	1.06	0.3	0.11	0.3	0.13	
O 1s	530.49	0.00	0.78	4.2	0.43	1.0	0.11	
N 1s	399.99	0.00	0.48	19.2	0.51	4.1	0.11	
Pb 4f7	137.89	0.00	4.76	10.2	0.17	31.9	0.20	I/Pb 2.86049
I 3d5	618.59	0.00	6.21	29.0	0.45	55.9	0.24	
C 1s	284.49	0.00	0.28	37.1	0.88	6.8	0.23	

* Chlorine (Cl) was also checked but not detected.

Table S4. Characteristic of perovskite solar cells of different blowing gas temperatures about reverse and forward scans of *J-V* measurements.

Sample	V_{OC} [V]	J_{SC} [mA/cm^2]	FF [%]	PCE [%]
HGB25 (Reverse)	1.13	23.13	75.28	19.70
HGB25 (Forward)	1.11	23.39	70.66	18.35
HGB75 (Reverse)	1.14	24.02	76.16	20.85
HGB75 (Forward)	1.13	24.02	72,12	19.53
HGB125 (Reverse)	1.07	23.85	74.92	19.20
HGB125 (Forward)	1.06	23.83	68.29	17.24

Table S5. Characteristic of perovskite solar cells of different blowing gas temperatures (HGB25, HGB75, and HGB125) about reverse and forward scans of *J-V* measurements, including parameters; *Voc, Jsc, FF*, and *PCE*. A total of 30 data in each condition are listed in the tables, and their average values are calculated at the bottom.

HGB25	V_{oc} [V]	J_{SC} [mA/cm^2]	Fill Factor [%]	PCE [%]
1	1.12	22.34	72.94	18.30
2	1.10	21.39	75.53	17.76
3	1.08	23.09	72.85	18.23
4	1.10	21.68	76.11	18.23
5	1.12	22.44	74.32	18.67
6	1.12	23.60	73.64	19.53
7	1.08	23.17	74.59	18.74
8	1.12	21.79	75.65	18.44
9	1.13	23.56	72.63	19.28
10	1.10	21.65	75.07	17.92
11	1.12	22.11	73.40	18.21
12	1.11	22.22	75.15	18.56
13	1.11	22.56	73.23	18.41
14	1.11	22.25	74.21	18.27
15	1.13	23.21	73.39	19.21
16	1.11	22.29	74.61	18.43
17	1.12	22.44	75.85	19.02
18	1.11	22.74	75.54	19.03
19	1.09	22.96	74.91	18.67
20	1.11	22.11	75.08	18.36
21	1.11	22.66	74.28	18.67
22	1.11	21.58	76.20	18.22
23	1.10	22.56	75.06	18.71
24	1.11	22.45	74.65	18.53
25	1.10	21.75	74.00	17.67
26	1.12	21.55	73.11	17.68
27	1.11	21.57	76.28	18.19
28	1.13	23.13	75.28	19.70
29	1.10	21.79	74.06	17.75
30	1.12	22.37	75.92	18.96
Average.	1.11	22.37	74.58	18.51

HGB75	V_{oc} [V]	J_{SC} [mA/cm^2]	Fill Factor [%]	PCE [%]
1	1.12	24.51	71.00	19.52
2	1.10	23.43	72.65	18.72
3	1.10	22.34	75.88	18.69
4	1.13	23.63	75.37	20.04
5	1.10	22.43	74.73	18.48
6	1.12	23.09	76.78	19.92
7	1.12	23.12	74.25	19.25
8	1.10	23.01	71.90	18.27
9	1.10	23.11	73.29	18.69
10	1.10	22.30	75.39	18.55
11	1.13	24.02	72.12	19.53
12	1.10	23.22	72.11	18.47
13	1.10	22.31	75.23	18.46
14	1.14	23.92	73.59	20.01
15	1.13	24.11	72.57	19.74
16	1.14	23.78	75.08	20.31
17	1.14	23.98	73.64	20.08
18	1.11	22.92	71.71	18.21
19	1.13	24.14	74.22	20.28
20	1.11	22.81	75.06	19.05
21	1.12	22.09	74.56	18.41
22	1.13	24.14	74.45	20.36
23	1.11	22.25	75.94	18.72
24	1.11	23.30	75.61	19.61
25	1.13	24.02	75.56	20.54
26	1.13	23.72	75.39	20.22
27	1.14	23.74	74.25	20.03
28	1.12	22.24	72.85	18.15
29	1.14	24.02	76.16	20.85
30	1.12	22.50	73.37	18.42
Average.	1.12	23.27	74.16	19.32

HGB125	V_{oc} [V]	J_{SC} [mA/cm^2]	Fill Factor [%]	PCE [%]
1	1.06	23.06	74.28	18.16
2	1.10	22.78	69.82	17.51
3	1.10	23.35	72.93	18.67
4	1.10	23.63	71.62	18.54
5	1.06	22.73	74.44	17.92
6	1.08	23.55	74.46	18.88
7	1.06	22.86	74.31	17.93
8	1.10	23.30	73.61	18.83
9	1.10	22.48	74.67	18.40
10	1.07	23.85	74.92	19.20
11	1.06	23.27	72.62	17.90
12	1.10	22.37	69.65	17.12
13	1.08	23.46	75.15	19.04
14	1.10	22.50	74.69	18.48
15	1.11	22.70	72.55	18.28
16	1.09	23.48	71.36	18.31
17	1.10	22.80	69.75	17.56
18	1.09	22.42	71.65	17.51
19	1.10	22.73	71.94	17.92
20	1.09	22.82	71.22	17.73
21	1.11	24.07	67.57	18.03
22	1.11	24.05	68.30	18.20
23	1.09	22.69	70.56	17.49
24	1.09	22.69	71.37	17.71
25	1.10	22.37	70.81	17.49
26	1.11	22.50	72.66	18.13
27	1.07	24.02	71.95	18.57
28	1.11	24.25	69.98	18.85
29	1.11	22.51	72.72	18.19
30	1.07	23.07	71.26	17.59
Average.	1.09	23.08	72.09	18.14
